# Thermochemical Activation of Wood with NaOH, KOH and H_3_PO_4_ for the Synthesis of Nitrogen-Doped Nanoporous Carbon for Oxygen Reduction Reaction

**DOI:** 10.3390/molecules29102238

**Published:** 2024-05-10

**Authors:** Galina Dobele, Aleksandrs Volperts, Ance Plavniece, Aivars Zhurinsh, Daina Upskuviene, Aldona Balciunaite, Gediminas Niaura, Luis César Colmenares-Rausseo, Loreta Tamasauskaite-Tamasiunaite, Eugenijus Norkus

**Affiliations:** 1 Latvian State Institute of Wood Chemistry, Dzerbenes Str. 27, LV-1006 Riga, Latvia; galina.dobele@kki.lv (G.D.); ance.plavniece@kki.lv (A.P.); aivarsz@edi.lv (A.Z.); 2 Center for Physical Sciences and Technology, Sauletekio Ave. 3, LT-10257 Vilnius, Lithuania; daina.upskuviene@ftmc.lt (D.U.); aldona.balciunaite@ftmc.lt (A.B.); gediminas.niaura@ftmc.lt (G.N.); loreta.tamasauskaite@ftmc.lt (L.T.-T.); 3 SINTEF Industry, Sustainable Energy Technology, Strindvegen 4, NO-7465 Trondheim, Norway; luis.colmenares-rausseo@sintef.no

**Keywords:** activated carbons, pyrolysis, hydrothermal treatment, nitrogen doping, ORR, fuel cells

## Abstract

Carbonization of biomass residues followed by activation has great potential to become a safe process for the production of various carbon materials for various applications. Demand for commercial use of biomass-based carbon materials is growing rapidly in advanced technologies, including in the energy sector, as catalysts, batteries and capacitor electrodes. In this study, carbon materials were synthesized from hardwood using two carbonization methods, followed by activation with H_3_PO_4_, KOH and NaOH and doping with nitrogen. Their chemical composition, porous structure, thermal stability and structural order of samples were studied. It was shown that, despite the differences, the synthesized carbon materials are active catalysts for oxygen reduction reactions. Among the investigated carbon materials, NaOH-activated samples exhibited the lowest Tafel slope values, of −90.6 and −88.0 mV dec^–1^, which are very close to the values of commercial Pt/C at −86.6 mV dec^–1^.

## 1. Introduction

Pyrolysis is a promising thermochemical technology for converting biomass into energy and chemical products. The demand for the commercial use of biomass-based carbon materials is growing rapidly in the field of modern technologies in medicine, environmental protection and energy as catalysts, battery and capacitor electrodes [1,2,3]. The fundamental reasons for this are both the renewability of precursors and the chemical composition and structure of carbons, which allow for an infinite variety of three-dimensional modifications that are capable of incorporating heteroatoms to form functional groups that determine the self-organization, chemical stability and reactivity of the synthesized materials.

The characteristics of activated carbons largely depend on the biomass pretreatment, the chemical properties of the activator and the conditions of the pyrolysis activation process. Activators determine the different pathways of raw material transformation, which lead to the synthesis of materials with different structures and properties; however, even being highly porous, these carbons do not always satisfy the required catalytic activity in specific reactions. Therefore, it is important to study both the conditions of pretreatment and pyrolysis, as well as the activation mechanisms.

The advantages of biomass chemical activation, as opposed to physical activation, are lower temperature and shorter pyrolysis time. The resulting products have sufficiently high yield, developed surface and controlled porosity [4]. The most commonly used chemical activators are H_3_PO_4_ [5,6,7,8], NaOH [4,9], KOH [10,11,12] and ZnCl_2_ [13], which are combined with various organic raw materials after carbonization or without it (for example, in the case of coal) and activated in an atmosphere of inert gases. Alkalis (KOH, NaOH) are the most promising activators [4], which react with carbon-containing precursors and form activated carbon (AC) with a developed porous structure and water-soluble salts that can be consequently washed in the course of demineralization. The mechanism of interaction of alkalis, mainly using KOH as an example, has been studied in sufficient detail [14,15]. Marsh et al. described the activation process of KOH [16]. The authors proposed two main reactions that promote chemical activation. The first is based on the catalysis of alkali metal reactions to form carbon oxide and carbon dioxide. The second is the reduction of hydroxide to free metallic potassium and its penetration between the carbon layers with lattice expansion by intercalated metal. Subsequently, the intercalate is removed from the carbon matrix. The reaction schemes proposed by other authors do not contradict each other. Otowa and others [17] believed that at temperatures below 700 °C, the main products are hydrogen, water, carbon monoxide, carbon dioxide, potassium oxide and carbonate. At temperatures above 700 °C, metallic potassium is formed in parallel. This process is associated with the formation of pores, which significantly reorganizes the organic spatial structure.

K_2_O + H_2_ → 2K + H_2_O (1)

K_2_O + C → 2K + CO (2)

Phosphoric acid is widely used for the production of activated carbon from lignocellulosic materials [18,19,20,21,22,23]. During thermal treatment, phosphoric acid reacts with the inner cell walls of lignocellulosic materials, turning biomass into a carbon material with a highly developed pore structure. Carbonaceous material is formed through hydrolysis and dehydration, followed by condensation (above 300 °C) [18,22,24]. At temperatures > 450° C, an increase in aromatic structures in the material is observed due to the loss of aliphatic, carboxyl and carbonyl groups [24]. Yagojen and Derbyshire [18] argue that phosphoric acid has two functions in this process. The first is the catalysis of cleavage reactions and the creation of cross-links through the processes of cyclization and condensation. The second is the formation of phosphate and polyphosphate bridges, which connect and cross-link biopolymer fragments. Using phosphoric acid for chemical activation leads to the production of activated carbons, which contain acidic phosphorus-containing groups [9,25]. Phosphate groups remain on the carbon surface even after demineralization of activated carbon [26,27].

The first step in the chemical activation of biomass is usually pre-carbonization using low-temperature pyrolysis or hydrothermal treatment [28]. Thanks to this stage, the barrier to degradation is reduced and there is an increase in pore volume and surface area, which have a beneficial effect on the subsequent interaction of raw materials with the activator [29]. Different carbonization conditions, as well as activation mechanisms, certainly affect the subsequent AC formation process and the properties of carbon material. When choosing an activator, it is necessary to take into account the carbonization conditions and the properties of the precursor. However, comparative data on the effect of lignocellulosic materials carbonization methods on the properties of carbon materials activated with various chemical agents are lacking. Carbonization of biomass residues followed by activation has great potential to become a safe process for producing various carbon materials for environmental, catalytic, electronic and agricultural applications. Recently, along with traditional thermal carbonization, a new type of hydrothermal carbonization (HTC), an environmentally friendly technology for the production of solid carbon material called hydrochar, has often been used. In recent years, HTC has attracted significant attention due to its ability to convert biomass wastes, including wet wastes, into carbon materials with useful characteristics, facilitating efficient use for adsorption, catalysis and porous carbon synthesis [30]. The difference in the chemical composition of biomass precursors after thermal and hydrothermal carbonization makes it possible to optimize activation processes to obtain the required pore distribution.

Among the numerous areas of use of activated carbons, energy conversion devices, including low-temperature fuel cells, play an important role [31]. The most active catalyst for the oxygen reduction reaction (ORR) is platinum, but its use is limited by high prices and dwindling resources [32]. Carbon-based catalysts are among the most promising due to their activity, developed porous structure, corrosion resistance, low cost and high electronic conductivity [33,34,35,36]. The oxygen reduction reaction at the cathode of a fuel cell proceeds slowly due to a strong O=O bond; therefore, to improve the kinetics of this reaction and catalytic properties, doping the structure of carbon materials with heteroatoms is used [37]. Nitrogen heteroatoms are introduced into the structure of carbon and transformed into surface functional groups under the influence of high-temperature and chemical treatment of precursors in order to improve the properties of materials for electrochemical applications. Electrochemical studies have proven that the pyridine-N, pyrrole-N and quaternary N forms have favourable electrochemical properties in oxygen reduction, comparable to those of commercial platinum-based electrode materials. Using these materials as electrodes in fuel cells may eliminate the use of noble metal electrodes. Doping with nitrogen, which has a higher electronegativity, leads to a redistribution of the charge of carbon atoms, generates a positive charge density on carbon atoms and changes the chemisorption of oxygen, which weakens the O=O bond [38,39].

The purpose of this work was to study the influence of the carbonization methods of birch wood and the application of three activators (phosphoric acid and potassium and sodium hydroxides) on the morphology, chemical composition and porous structure of the synthesized activated carbons. The resulting nitrogen-doped carbons were tested as alternative oxygen reduction catalysts to commercial platinum/carbon composite using the disk electrode rotating system.

With this work, we aimed to promote the optimal choice of biomass pretreatment method and activator to optimize the structure of carbon materials for use as oxygen reduction catalysts.

## 2. Results

### 2.1. Characteristics of Chemical Composition and Structure of Carbon Materials

In this work, properties of activated carbons based on birch wood (*Betula pendula*) precursors, carbonized using two methods, namely pyrolysis at 500 °C (samples denoted with prefix AWC) and hydrothermal treatment at 250 °C (samples denoted with prefix AHTC), were studied. The carbonized materials were activated using three chemical compounds: potassium (samples denoted with suffix K) and sodium (samples denoted with suffix Na) hydroxides and phosphoric acid (samples denoted with suffix P). Activation using various activators was carried out to compare their effectiveness in the formation of a porous structure, as well as to determine the specific influence of the porous structure on the catalysis of the oxygen reduction reaction.

Chemical activation of wood with phosphoric acid and alkali metal hydroxides is effective in providing the required properties of carbon structure (e.g., high specific surface area, developed micro- and mesoporosity); however, in both cases, there are also negative aspects. The main disadvantages were, in the case of acid, corrosion of metal equipment; and in the case of hydroxides, significant amounts of wastewater during demineralization with a fairly high concentration of alkali metal salts were produced. Therefore, when choosing an activator, it is important to evaluate the properties of activated carbon and the complexity of the production process.

Authors of studies regarding the activation process with potassium and sodium hydroxides usually point to a similar interaction mechanism with organic raw materials [40,41]. However, the bulk of published work is devoted to KOH studies, although, in comparison with NaOH, this hydroxide has higher molecular weight, higher melting point and higher price and is, therefore, less economically feasible, while being more environment friendly, since it potentially can be used as fertilizer in agriculture. The mass ratio of the activator to the carbonized material when using H_3_PO_4_ and NaOH was 3:1. In the case of KOH, to preserve the molar ratio for comparison to sodium hydroxide, it was 4.2:1.

The results of the elemental analysis of the activated samples, presented in Table 1, show that in the series of activators H_3_PO_4_-KOH-NaOH the carbon content increases and the oxygen content decreases. It should be noted that, regardless of the carbonization method, the lowest oxygen content (about 4%) is observed when NaOH was used, regardless of the carbonization method, which is almost twofold less than that in case of KOH, indicating the presence of different porous structures. A possible explanation for the differences in oxygen content in the structure of alkali-activated carbons is the steric factor. The smaller ionic radius of sodium cations, compared to the radius of potassium ions, obviously allows them to more actively intercalate into the layers of the precursor to interact with oxygen-containing groups. The influence of various carbonization methods has little effect on the elemental composition of alkali-activated carbon. When H_3_PO_4i_ is used as an activator of hydrothermally obtained carbonizate, the highest oxygen content (14.15%) and the lowest carbon content (82.01%) are observed due to the introduction of phosphate groups.

**Figure 1:**
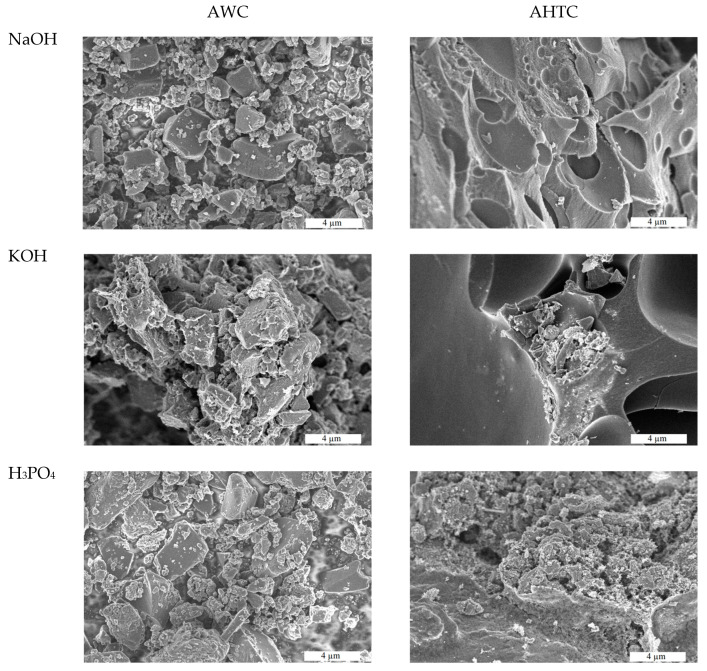
SEM images of activated carbons based on pyrolyzed and hydrothermally carbonized wood (scale 4 µm).

As can be seen in the SEM images (Figure 1), both the activator and the pretreatment of the raw materials affect the shape and size of the particles. When pyrolyzed wood is used as a raw material, the agglomerated particles are smaller—5–20 µm, and when hydrothermal carbonization is used, they are larger—40–60 µm. It should also be noted that in the case of hydrothermally carbonized wood activated by NaOH, less-dense lamellar particles are formed. Features of the morphology of samples pretreated pyrolytically and hydrothermally possibly influence the subsequent activation process and the formation of a porous structure.

**Figure 2:**
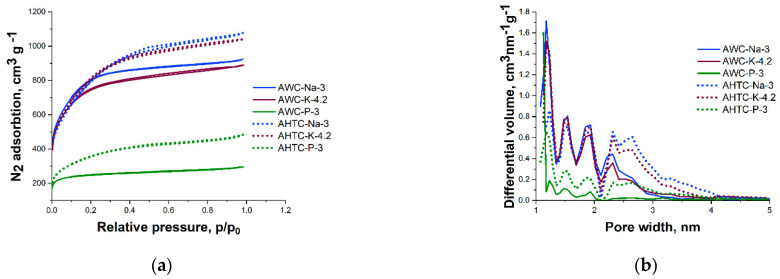
N_2_ adsorption–desorption isotherms (**a**) and pore size distribution (**b**) of activated carbon with hydroxides (NaOH, KOH) and H_3_PO_4_ from pyrolyzed (straight line) or hydrothermal carbonized wood (dashed line).

Based on nitrogen sorption at 77K isotherms, it can be determined that the more developed porous structure is formed after activation with hydroxides. The specific surface area upon activation with hydroxides calculated according to BET theory exceeds the theoretical maximum, and the total pore volume is more than 1.6 cm^3^ g^−1^ (Table 2). In the case of activation with NaOH, a slightly higher nitrogen sorption is observed compared to that with KOH, and the specific surface area reaches 2892 m^2^ g^−1^ for hydrothermal carbonization and 2909 m^2^ g^−1^ for pyrolysis (Table 2). Samples carbonized hydrothermally for all activators adsorb more nitrogen and, as demonstrated by pore size distribution (Figure 2, right), also have wider mesopores.

The porosity of activated H_3_PO_4_ samples is lower compared to hydroxides, regardless of the carbonization method (S_BET_ for hydrothermal carbonization and pyrolysis, 1739 and 769 m^2^ g^−1^, respectively) (Table 2), and the sample after carbonization by pyrolysis is practically microporous: the micropore volume is 0.34 with a total volume of 0.41 cm^3^ g^−1^. In the case of hydrothermal pretreatment (sample AHTC-P-3), the proportion of mesopores in the total pore volume increases up to 43% compared to the sample pretreated pyrolytically—17% for AWC-P-3, respectively (Table 2, Figure 2).

**Figure 3:**
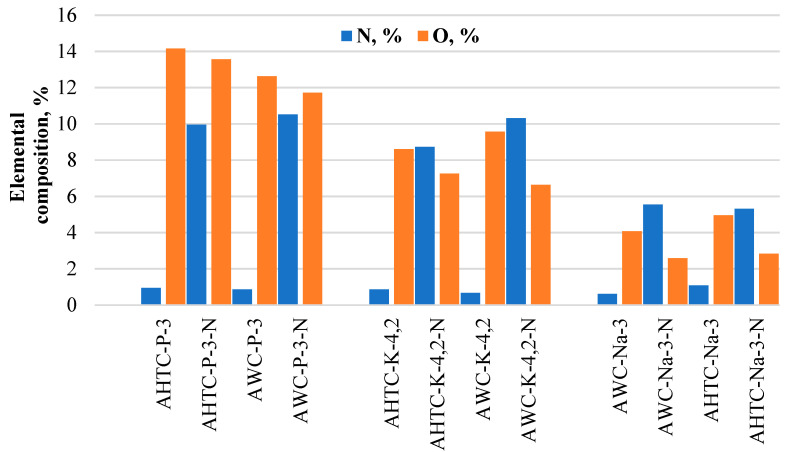
Oxygen and nitrogen content of activated carbon (activators H_3_PO_4_, KOH and NaOH) from pyrolyzed (AWC) or hydrothermally carbonized (AHTC) wood before and after N-doping.

Nitrogen doping was used to increase the efficiency of the resulting carbon materials in terms of oxygen reduction reaction catalysis, providing not only a decrease in the content of elemental oxygen in the structure but also a redistribution of charge on carbon atoms, since nitrogen has a higher electronegativity, thereby creating a positive charge density on carbon atoms and promoting ORR [42]. The data presented in Table 1 and Figure 3 show that the content of introduced nitrogen in doped samples is related to the oxygen content. The highest nitrogen content is in carbon materials activated with H_3_PO_4_ and KOH (about 8–10%) and the lowest (about 5%) is in samples activated with NaOH with the lowest oxygen content in undoped samples.

On the other hand, it should be noted that in samples of activated NaOH, after doping with nitrogen, the oxygen content decreases more than twofold (from 5 to 2%) (Table 1, Figure 3). For samples activated with H_3_PO_4_ and doped with nitrogen, despite the high nitrogen content, a slight decrease in the oxygen content is observed (the difference is about 1%). The presence of residual oxygen in these samples is apparently due to phosphorus-containing ester groups. However, similar patterns of changes in elemental oxygen are also observed for hydrothermally carbonized and activated KOH samples. For a precursor carbonized by pyrolysis and activated with KOH, after doping, the oxygen content decreases from 9.58 to 6.63%. When KOH is used for the activation of sample after hydrothermal carbonization, the decrease in oxygen content is only 1.34%.

**Figure 4:**
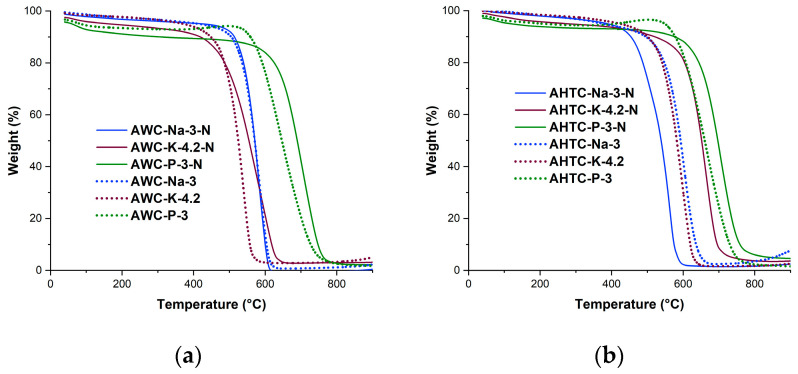
TG curves of carbon materials pretreated with carbonization by pyrolysis (AWC) and hydrothermal treatment (HTC) and activated with H_3_PO_4,_ NaOH, KOH; (**a**) before and (**b**) after doping with nitrogen.

Doping with nitrogen changes the thermal stability of all the resulting activated carbon materials differently (Figure 4). H_3_PO_4_-activated samples, regardless of the carbonization method, having the greatest thermal stability before doping, also remain the most stable after doping, possibly due to the fact that these samples have the most condensed structure, which is also reflected in the lower porosity of these materials, as well as the presence of stable phosphorus bonds. The carbonization method and doping process have little effect on the stability of the NaOH-activated sample. The KOH-activated sample becomes more stable as a result of doping, with the greatest stability observed in the hydrothermally carbonized sample.

This may be due to the fact that under doping conditions at 800 °C, additional stable C-C chemical bonds appear in the structure of this carbon material due to structural fragments that were difficult to access by KOH during activation at 700 °C. Thus, the different thermal stability of the samples activated by NaOH and KOH also indicates differences in their structure, which later will be exemplified and explained by the degree of amorphization, as shown by Raman spectroscopy.

**Figure 5:**
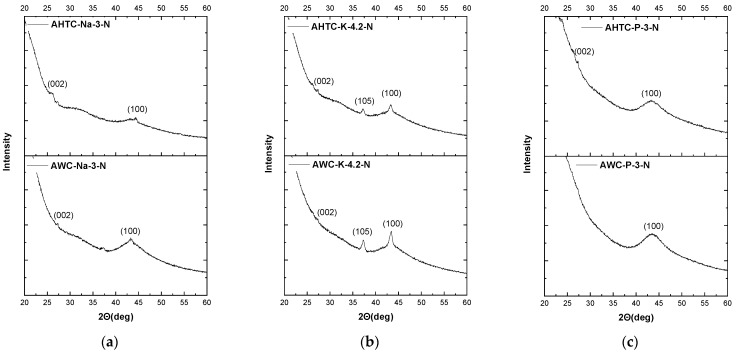
XRD patterns of N-doped activated carbon samples: NaOH activation (**a**); KOH activation (**b**); H_3_PO_4_ activation (**c**).

Figure 5 shows the XRD patterns of N-doped activated carbon samples under study, where two peaks at 2Θ = ~25 and ~43 can be assigned to typical graphitic (002) and (100) planes, respectively. Additional peaks can be observed in the case of KOH-activated samples (Figure 5b) at 2Θ = ~24 and according to ICSD#04-0345, these can be related to the Miller index (105) of residual potassium.

**Figure 6:**
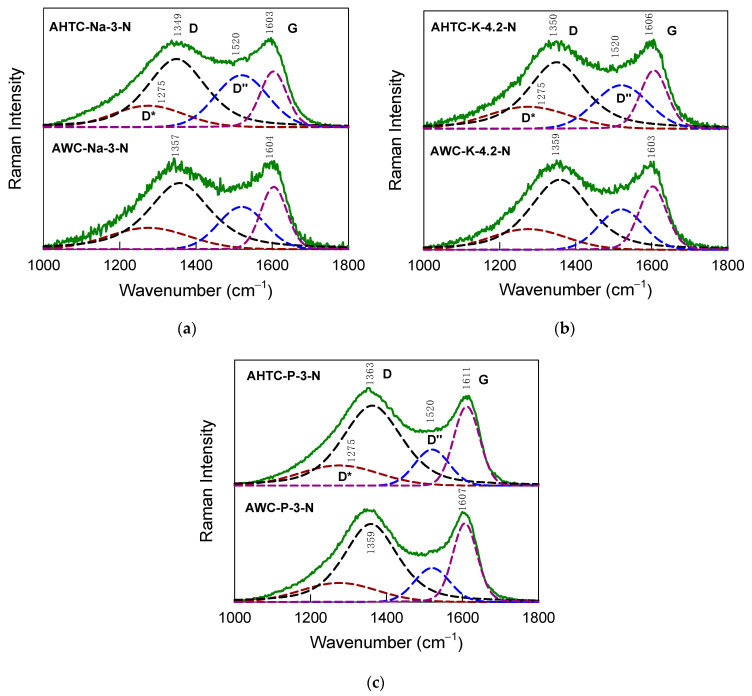
Raman spectra of carbon materials obtained by carbonization by pyrolysis (AWC) and hydrothermal treatment (AHTC) activated with (**a**) NaOH, (**b**) KOH and (**c**) H_3_PO_4_.

Figure 6 compares 532-nm excited Raman spectra of samples AHTC-Na-3-N (a), AWC-Na-3-N (a), AHTC-K-4.2-N (b), AWC-K-4.2-N (b), AHTC-P-3-N (c) and AWC-P-3-N (c). All the spectra exhibit broad D and G bands. No clearly defined features at 2688–2694 cm^–1^ were observed in the case of any samples. This type of feature belongs to the prominent 2D band, and the absence of a clearly defined band indicates a high degree of disordering in the studied samples [43].

Important quantitative information about the structure of carbon material can be extracted from the analysis of full width at half the maximum of the G band, FWHM(G) [44,45,46,47]. Therefore, the Raman spectra in the frequency range from 1000 to 1800 cm^–1^ were fitted by 4 Gaussian or Lorentzian–Gaussian form components (Figure 6). Besides the well-known D and G components (Lorentzian–Gaussian form), additional Gaussian form bands located at 1275 cm^–1^ (D*) and 1520 cm^–1^ (D″) were introduced [44,45,46,47,48]. The D” band was previously related to the amorphization of carbon material [49,50]. This feature may also contain contributions from the C=C vibrations of polyenes [51,52]. The broad band in the frequency region of 1220–1270 cm^–1^ (similar to our D* band) in the Raman spectra of nanoporous activated carbon material was previously assigned to the amorphous carbon-related D band and C–C vibrations of cis-polyacetelyne groups [50].

The important structural parameter in sp^2^-hybridization layered carbon materials is its average in-plane crystallite size, *L*_a_ [44,46]. The experimentally obtained FWHM(G) value can be used to determine the *L*_a_ Equation (3) [44,47,53]:

(3) $$L_{a}=\frac{l_{c}}{2}ln\left[\frac{C}{FWHM\left(G\right)-FWHM(G_{0})}\right]$$

where photon coherence length *l*_c_ = 32 nm, *C* = 95 cm^–1^ and FWHM(G) and FWHM(G_0_) are the widths of the G band of the sample under investigation and undoped pristine graphene (15 cm^–1^), respectively. The equation is valid for measuring the L_a_ between 32 and 2.8 nm [44]. The FWHM(G) and estimated L_a_ values are given in Table 3. The La varies from 4.7 to 7.1 nm depending on the sample treatment. The largest in-plane crystallite size values were obtained for the AWC-P-3-N sample. Another important structural parameter is the relative intensity of D″ band measured as intensity ratio *I*(D″)/*I*(G) [54]. This parameter probes the amorphization of carbon material. The highest ratio was found for the AHTC-Na-3-N sample, indicating the highest amount of amorphous phase in this material.

**Figure 7:**
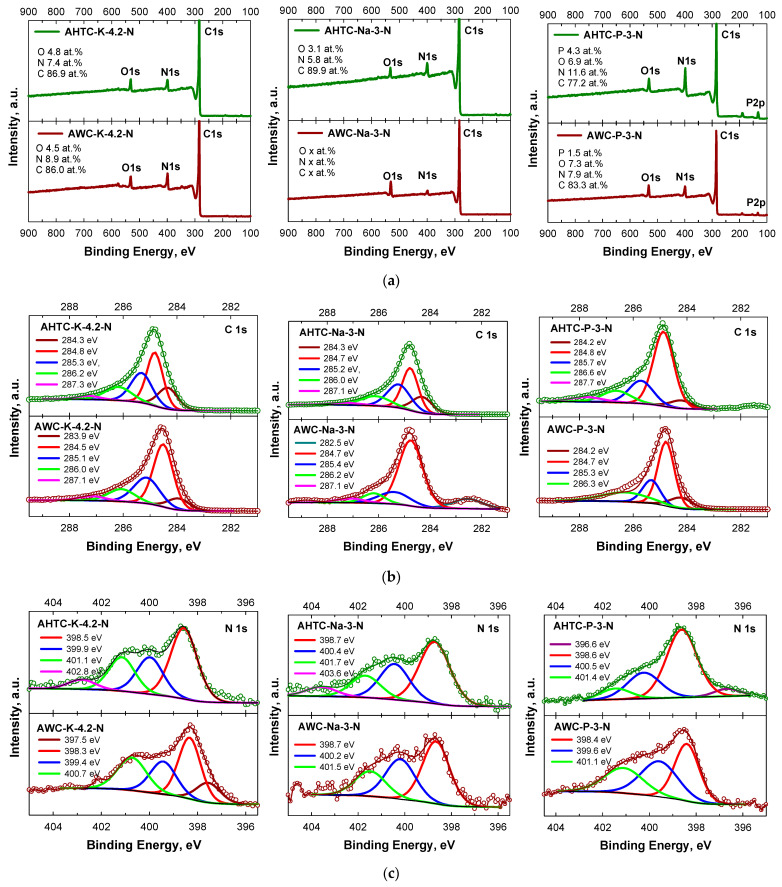

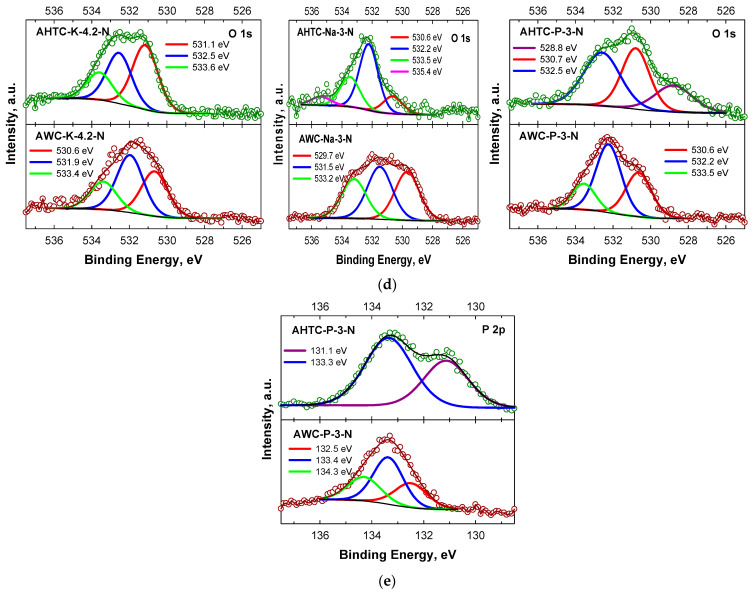
XPS (**a**) survey, (**b**) C 1s, (**c**) N 1s spectra, (**d**) O 1s and (**e**) P 2p spectra of investigated carbon materials.

XPS was used to further analyse the chemical elemental composition of the surface of the carbon materials under study (Table 4, Figure 7). The survey spectra of all samples obtained in Figure 7a show the presence of the elements C, O and N elements for all carbon samples and additionally P for AHTC-P-3-N and AWC-P-3-N (Figure 7e).

The high-resolution XPS C 1s spectra of x samples are divided into four major peaks, occurring at 283.9–284.3 (Csp^2^), 284.5–284.8 (Csp^3^), 285.1–285.7 (N-sp^2^-C) and 286.0–286.6 (N-sp^3^-C) [55] (Figure 7b).

Deconvolution of high-resolution N 1s spectra shows that most of the nitrogen is in catalytically active pyridinic N form (Figure 7c, Table S1)—the N 1’s peak was fitted and the peaks were identified as pyridinic-N (398.5 eV), pyrrolic-N (400.1 eV) and quaternary nitrogen, an N atom with a formal charge of +1, e.g., (protonated) pyridinic-N (401.1 eV), as well as smaller peaks of protonated pyrrole (402.8 eV).

The O1s’ spectra for all samples show peaks with binding energies of 528.8–530.7, 532 ± 0.5 and 533.5 ± 0.1 eV (Figure 7d). Typically, the peaks at the lower binding energy of 528–531 eV indicate the presence of the lattice oxygen species [56], whereas the O1s’ peaks located at 532 ± 0.5 and 533.5 ± 0.1 eV can be associated with oxygen in the states of C=O and C–OH/C–O–C, respectively [56].

P 2p XPS spectra for the two samples activated using phosphorous acid (namely AWC-P-3-N and AHTC-P-3-N) (Figure 7e) reveal the following peaks and corresponding states 133.2 eV (PO_4_)^3−^—phosphate, 134.3 eV—H_3_PO_4_ and 132.5 eV Ph_3_PO (triphenylphosphine oxide).

The ratio of each state of C, O, N and P atoms in atomic % is demonstrated in Table S1 (supplementary data).

### 2.2. Catalytic Properties of Nitrogen-Doped Carbon Materials

**Figure 8:**
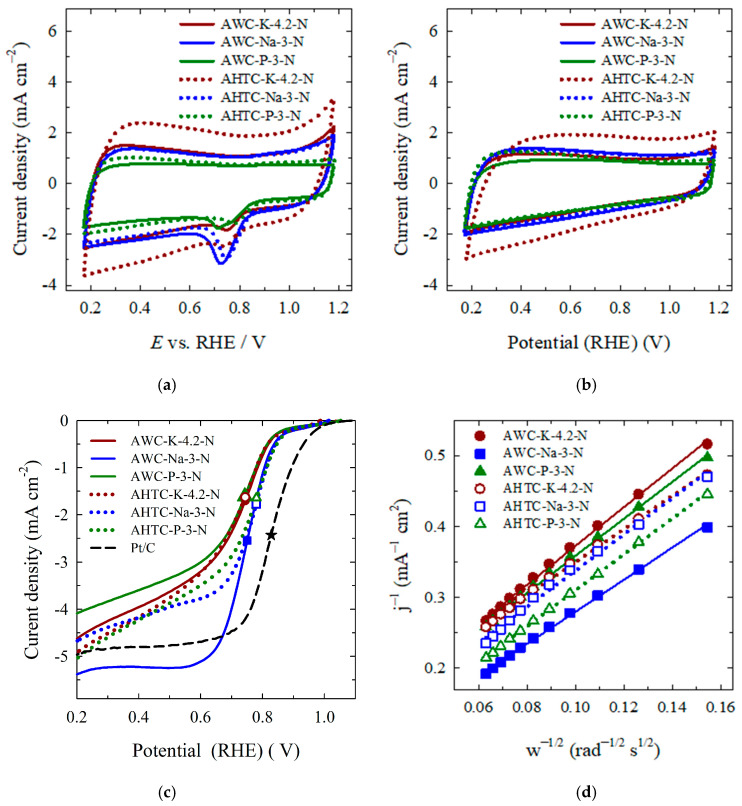
CVs recorded from carbon materials in an O_2_-saturated 0.1 M KOH solution (**a**) and Ar-deaerated 0.1 M KOH solution (**b**) at 10 mV s^–1^. (**c**) LSVs recorded from carbon materials in O_2_-saturated 0.1 M KOH solution at 10 mV s^–1^ at 1600 rpm. The symbols show the *E*_1/2_ values for each catalyst. (**d**) Koutecky–Levich plots at electrode potential 0.20 V.

Since it was previously shown by the authors of this paper that N-doping has a decisive influence on activated carbon materials’ electroactivity for ORR [2], only nitrogen-enriched samples were investigated for oxygen reduction. Figure 8a shows the CVs of carbon materials in O_2_-saturated 0.1 M KOH solution at a scan rate of 10 mV s^–1^. For comparison, CVs were also recorded from the carbon materials in an Ar-deaerated 0.1 M KOH solution (Figure 8b). No distinct redox peaks are observed in an Ar-deaerated 0.1 M KOH solution, whereas apparent reduction peaks are seen in the CVs recorded from carbon materials at approximately 0.71 ± 0.3 V in an O_2_-saturated 0.1 M KOH solution, indicating the activity of carbon materials for ORR (Figure 8a).

ORR performance was also investigated using a rotating disk electrode by recording LSVs in O_2_-saturated 0.1 M KOH solution at rotation speeds from 400 to 2400 rpm at 10 mV s^–1^. A comparison of ORR LSVs recorded from carbon and commercial Pt/C catalysts is shown in Figure 8c. The onset potential (*E*_onset_) was marked as the potential at an ORR current density of –0.1 mA cm^–2^. The synthesized carbon materials are active ORR catalysts in an alkaline medium, showing an *E*_onset_ of 0.94 ± 0.3 V and a corresponding half-wave potential (*E*_1/2_) of approximately 0.75 ± 0.3 V, which are close to the *E*_onset_ and *E*_1/2_ values for the commercial Pt/C catalyst, i.e., 0.99 and 0.83 V, respectively (Figure 8c, Table 5).

**Figure 9:**
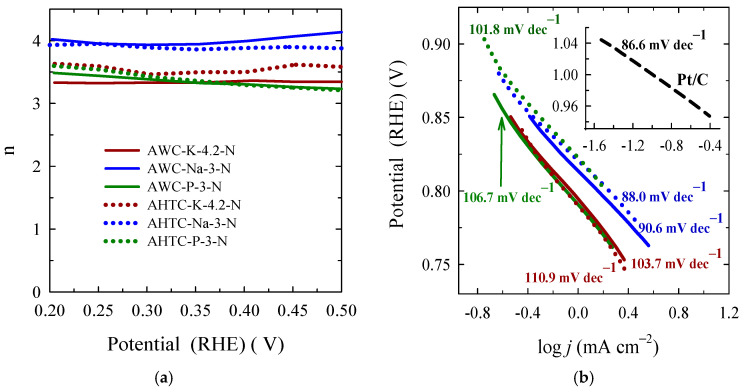
Number of electrons transferred (**a**) and corresponding Tafel slopes for carbon materials (**b**) and Pt/C in the inset.

The number of electrons transferred per O_2_ molecule (n) was calculated using the Koutecky–Levich (K-L) Equations (4)–(6) [71]:

(4) $$\frac{1}{j}=\frac{1}{j_{k}}+\frac{1}{j_{d}}=\frac{1}{B\omega^{1/2}}+\frac{1}{j_{k}}$$

(5) $$B=0.62nFC_{0}D_{0}^{2/3}\nu^{-1/6}$$

(6) $$j_{k}=nF{kC}_{0}$$

where j, j_k_ and j_d_ are the experimentally measured current density, kinetic and diffusion-limiting current densities, respectively; k is the electrochemical rate constant for O_2_ reduction, F is the Faraday constant (96,485 C mol^–1^), C_0_ is the concentration of oxygen in bulk (1.2 × 10^–6^ mol cm^–3^), D_0_ is the diffusion coefficient of O_2_ (1.9 × 10^–5^ cm^2^ s^–1^), ν is the kinematic viscosity of the solution (0.01 cm^2^ s^–1^), and ω (rad s^–1^) is the rotation rate of the electrode [72]. The Koutecky–Levich (K-L) plots of the carbon catalysts at 0.2 V potentials show good linearity, allowing for the determination of electron numbers transferred during the ORR process (Figure 8d). Furthermore, a linear relationship between j^–1^ and ω^–1/2^ indicates the first-order dependence of the ORR kinetics at varied potentials (0.20–0.50 V). Figure 9a reveals the number of electrons transferred at various potentials at the carbon and Pt/C catalysts. A value of n close to 4 on all studied potentials (Figure 9a, Table 5) was obtained on AWC-Na-3-N and AHTC-Na-3-N catalysts, indicating the direct 4e^−^ reduction pathway from O_2_ to H_2_O predominantly.

The Tafel analysis was carried out using the empirical Tafel equation Equation (7), which is based on a linear region between the logarithms of measured current density and the applied potential in order to evaluate the ORR activity of the synthesized N-doped carbon materials:

(7) ηηη=a+blogj

where η (V) is the applied overpotential, a (V) is the curve intercept, b (V dec^–1^) is the Tafel plot, and j (A cm^–2^) is the resulting current density. A smaller Tafel slope means the low overpotential needs to reach a high current density. Figure 9b presents the mass-transfer corrected Tafel plots using the Koutecky–Levich equation, where the kinetic current density for ORR (j_K_) could be calculated according to the Equation (8):

(8) $$j_{k}=\frac{j_{L}j}{j_{L}-j}$$

where j_L_ is the O_2_-diffusion-limited current density at 0.2 V vs. RHE measured at 1600 rpm and j is the Faradaic current density. The obtained Tafel slope values for all carbon materials and Pt/C (the inset) are given in Figure 9b. Among the investigated pyrolyzed and hydrothermally carbonized carbon materials, the AWC-Na-3-N and AHTC-Na-3-N exhibits the lowest Tafel slope values of −90.6 and −88.0 mV dec^–1^, respectively, which are very close to the commercial Pt/C (−86.6 mV dec^–1^, Figure 9b inset). The higher catalytic activity of NaOH activated carbon materials can be related to the combination of the number of properties, such as ratio of micro- and mesopores, degree of graphitisation and content of heteroatoms induced active centres, which synergistically promote their efficiency towards ORR.

## 3. Materials and Methods

### 3.1. Synthesis of Materials

In this work, birch wood chips (fraction 4–6 mm), which were carbonized before activation (at 500 °C in an inert atmosphere or hydrothermally at 250 °C), were used. The ground carbonizates were mixed with the activators and treated at 700 °C for 2 h under a flow of argon, then the resulting material was washed with demineralized water. After activation with alkalis, it was then treated with hydrochloric acid (Sigma Adrich, Burlington, MA, USA, >99%) (10%) and washed with water to pH 5. The following mass ratios were used:

H_3_PO_4_ (Sigma Adrich, >99%), to carbon 3:1
KOH (Honeywell Fluka, Puriss. p.a., Charlotte, NC, USA, >98%), to carbon 4.2:1
NaOH (Honeywell Fluka, Puriss. p.a., >98%), to carbon 3:1

The ratios of KOH (56.1 g mol^−1^) and NaOH (40.0 g mol^−1^) were chosen to preserve the molar ratio of activator addition.

The obtained material was mixed with dicyandiamide (DCDA, Sigma Adrich, >99%) solution in dimethylformamide (DMF, Honeywell Fluka, ACS reagent, >98.8%) in mass ratio 1 to 20, treated in a rotary evaporator at 50 mbar for one hour, and then the DMF was removed and carbons were doped with nitrogen at 800 °C for one hour in the argon flow.

### 3.2. Characterization of Synthesized Materials

Carbon, nitrogen and hydrogen content was evaluated using the Vario Macro CHNSO (Elementar Analysensysteme GmbH, Langenselbold, Germany) device. The oxygen content was calculated from the difference of 100%.

The porous structure (specific surface area, total volumes of micro- and mesopores and pore size distribution) was determined from isotherms of low-temperature adsorption–desorption of nitrogen at 77 K on Nova 4200e device (Quantachrome, Boynton Beach, FL, USA).

The morphology and composition of the prepared materials were investigated using a SEM/FIB workstation Helios Nanolab 650 (FEI, Eidhoven, The Netherlands) with an energy dispersive X-ray (EDX) spectrometer INCA Energy 350 X-Max 20 (Oxford Instruments, High Wycombe, UK).

Thermogravimetric analysis (TGA) was performed using TA Instruments Discovery (Waters, New Castle, DE, USA) at a rate of 10 °C/min in the range from 20 to 900 °C in air flow.

X-ray diffraction analysis (XRD) was measured using a D2 PHASER (Bruker, Karlsruhe, Germany) diffractometer and Cu-K-alpha as an X-ray source. The measurements were conducted in the 2θ range of 10°–90°.

Raman spectra were recorded using an inVia Raman (Renishaw, New Mills, UK) spectrometer equipped with a thermoelectrically cooled (−70 °C) CCD camera and microscope. Raman spectra were excited with 532 nm radiation from a pumped solid state (DPSS) laser (Renishaw, UK). The 20×/0.40 NA objective lens and 1800 lines/mm grating were used to record the Raman spectra. The accumulation time was 40 s. To avoid damaging the sample, the laser power at the sample was restricted to 0.4 mW. The Raman frequencies were calibrated using the polystyrene standard. Parameters of the bands were determined by fitting the experimental spectra with Gaussian and Lorentzian shape components using GRAMS/A1 8.0 (Thermo Scientific, East Grinstead, UK) software.

X-ray photoelectron spectroscopy (XPS) was used to analyse the chemical composition of the samples using a Kratos AXIS Supra+ spectrometer (Kratos Analytical, Manchester, UK) with monochromatic Al Kα (1486.6 eV) X-ray radiation powered at 225 W. The base pressure in the analysis chamber was less than 1 × 10^−8^ mbar, and a low-electron flood gun was used as a charge neutralizer. The survey spectra for each sample were recorded at a pass energy of 80 eV with a 1 eV energy step and high-resolution spectra (pass energy—10 eV, in 0.1 eV steps) over individual element peaks. The binding energy scale was calibrated by setting the adventitious carbon peak at 284.8 eV. XPS data were converted to VAMAS format and processed using the software program Avantage 6.7.0.86 (Thermo Scientific, East Grinstead, UK).

### 3.3. Electrochemical Measurements of Synthesized Materials

All electrochemical measurements were performed using the electrochemical software Nova 2.1.6 with a Metrohm Autolab potentiostat (PGSTAT100) (Metrhom, Herisau, Switzerland). A three-electrode electrochemical cell was used for electrochemical measurements. The working electrode was a glassy carbon (GC) electrode 5 mm in diameter. The Pt sheet was used as a counter-electrode, and Ag/AgCl in 3 M KCl was used as a reference electrode. The measurements were performed in an O_2_-saturated 0.1 M KOH solution.

Before the experiments, the GC electrode was polished. The catalyst ink was obtained according to the following steps: First, the 21.5 mg of the obtained catalyst was dispersed ultrasonically for 2 h in 457.5 μL ethanol, 475.5 μL deionized water and 85 μL 5 wt.% Nafion. Then, 8 μL of the prepared suspension mixture was pipetted onto the GC electrode with a geometric area of 0.196 cm^2^ and dried at room temperature overnight. The catalyst loading was 876 μg cm^−2^.

Linear-sweep voltammograms (LSVs) were recorded with a scan rate of 10 mV s^−1^ at rotation rates (ω) from 100 to 2400 rpm in an O_2_-saturated 0.1 M KOH solution. The data were collected at 100, 400, 800, 1200, 1600, 2000 and 2400 rpm. The electrode potential was quoted versus the reversible hydrogen electrode (RHE). The presented current densities are normalized to the geometric area of catalysts.

For comparison, commercial Pt/C catalyst (Tanaka, Tokyo, Japan, 46.4 wt.% Pt) with a catalyst loading of 80.9 μg cm^–2^ was used.

## 4. Conclusions

In this work, wood-based carbon materials were synthesized in sequence, using pre-carbonization under various conditions (pyrolysis at 500 °C or hydrothermal carbonization at 250 °C), thermochemical activation method (700 °C, 2 h) with KOH, NaOH and H_3_PO_4_ and then doping with nitrogen.

It has been shown that the indicators of elemental analysis, porous structure, thermal stability, crystallite size and degree of amorphization (Raman spectra) of carbon materials are influenced by both the carbonization method and the activator used, as well as the doping process.

The highest indicators of porous structure were obtained when using alkalis (S_BET_ more than 2000 m^2^ g^−1^), which are characterized by a bimodal distribution of pore sizes in the micro- and mesoporous structure. Samples activated by H_3_PO_4_ are characterized by a microporous structure with a smaller specific surface area. It has been shown that pore volume can be controlled using various carbonization methods; the contribution of mesopores increases during hydrothermal carbonization for all activators.

Nitrogen doping reduces the microporosity of samples and the oxygen content, which is most pronounced during activation using NaOH.

A comparison of the structure of carbon materials using the Raman scattering method showed that the largest crystallite sizes are observed in samples of activated H_3_PO_4,_ and the highest degree of amorphization is observed when activated with NaOH. The catalytic activity of the synthesized carbon materials was assessed in the reaction of oxygen reduction in 0.1 M KOH solution using the rotating disk electrode method. Despite the differences in performance, all of the resulting nitrogen-doped carbon materials showed high electrocatalytic activity in oxygen reduction, comparable to commercial catalysts containing Pt. The number of electrons transferred per O_2_ molecule is close to 4.0. A comparison of the properties of carbon materials activated by alkalis showed that the superior activity of carbon materials activated by NaOH compared to KOH, despite the similar chemical mechanism of interaction of alkalis, may be associated with a steric factor, namely, the smaller ionic radius Na + cation, allowing its deeper intercalation to precursor the structure and interaction with oxygen groups, creating active centres. The obtained results of the study of carbon materials are aimed at facilitating the development and correct selection of methods for carbonization of wood and choice of activator to optimize the structure of carbon materials for use as oxygen reduction catalysts.

## Figures and Tables

**Table 1:** Elemental composition of activated carbon (activators H_3_PO_4_, KOH and NaOH) from pyrolyzed (AWC) or hydrothermally carbonized (AHTC) wood before and after N-doping.

Sample	Elemental Composition				Ash, %
	N, %	C, %	H, %	* O, %	
AHTC-P-3	0.95	82.01	2.89	14.15	1.9
AHTC-P-3-N	9.95	73.51	2.98	13.56	3.7
AWC-P-3	0.86	83.61	2.91	12.62	2.3
AWC-P-3-N	10.52	74.73	3.04	11.71	2.8
AHTC-K-4.2	0.86	89.49	1.05	8.60	1.7
AHTC-K-4.2-N	8.73	81.32	2.69	7.26	2.7
AWC-K-4.2	0.67	88.88	0.88	9.57	2.0
AWC-K-4.2-N	10.31	80.24	2.82	6.63	1.8
AWC-Na-3	0.62	94.85	0.46	4.07	1.7
AWC-Na-3-N	5.55	90.97	0.89	2.59	1.2
AHTC-Na-3	1.09	93.3	0.65	4.96	1.5
AHTC-Na-3-N	5.31	90.05	1.81	2.83	1.4

* oxygen content is calculated in % based on the difference of 100% − (H% + C% + N%).

**Table 2:** Porous structure parameters of activated carbon (activators H_3_PO_4_, KOH and NaOH) from pyrolyzed (AWC) or hydrothermal carbonized (AHTC) wood before and after N-doping.

Samples	Specific Surface Area (BET), m^2^ g^−1^	Pore Volume, cm^3^ g^−1^			Mesopores from Vt, %
		Total (Vt)	micro *	meso **	
AHTC-P-3	1739	0.73	0.41	0.32	43
AHTC-P-3-N	921	0.55	0.32	0.23	42
AWC-P-3	769	0.41	0.34	0.07	17
AWC-P-3-N	520.9	0.28	0.22	0.06	21
AHTC-K-4.2	2926	1.61	0.96	0.65	40
AHTC-K-4.2-N	2029	1.039	0.66	0.38	36
AWC-K-4.2	2662	1.38	0.93	0.45	32
AWC-K-4.2-N	1679	0.82	0.583	0.24	29
AWC-Na-3	2909	1.67	0.92	0.74	45
AWC-Na-3-N	2497	1.34	0.86	0.48	36
AHTC-Na-3	2892	1.43	0.97	0.46	32
AHTC-Na-3-N	2521	1.289	0.831	0.46	36

* Volume of micropores was calculated using Dubinin–Radishkevich theory. ** Volume of mesopores is the difference between the total and micropores volumes.

**Table 3:** G-peak full width at half-maximum FWHM(G), average crystallite size *L*_a_ and intensity ratio *I*(D″)/*I*(G) of carbon-based samples.

Sample	FWHM(G) (cm^–1^)	*L*_a_ (nm)	*I*(D″)/*I*(G)
AHTC-Na-3-N	82.2	5.5	0.93
AWC-Na-3-N	81.5	5.7	0.68
AHTC-K-4.2-N	84.6	5.0	0.74
AWC-K-4.2-N	85.7	4.7	0.64
AHTC-P-3-N	80.7	5.9	0.46
AWC-P-3-N	76.1	7.1	0.43

**Table 4:** Determination of surface elemental composition of carbon samples under study using XPS.

Element	AWC-K-4.2-N	AWC-Na-3-N	AWC-P-3-N	AHTC-K-4.2-N	AHTC-Na-3-N	AHTC-P-3-N
	at. %	at. %	at. %	at. %	at. %	at. %
N	8.9 ± 0.1	3.6 ± 0.1	7.9 ± 0.1	7.4 ± 0.1	5.8 ± 0.1	11.6 ± 0.1
O	4.5 ± 0.1	10.6 ± 0.1	7.3 ± 0.1	4.8 ± 0.1	3.1 ± 0.1	6.9 ± 0.1
C	86.0 ± 0.1	85.8 ± 0.1	83.3 ± 0.1	86.9 ± 0.1	89.9 ± 0.1	77.2 ± 0.1
P	-	-	1.5 ± 0.1	-	-	4.3 ± 0.1

**Table 5:** The data of onset potential, half-wave potential, the average number of electrons transferred per O_2_ molecule (n) and Tafel plots for samples under study and literature data for various catalysts for comparison.

Sample	*E*_onset_, V	*E*_1/2_, V	Average Number of Electrons Transferred, n	Tafel Slope, mV dec^–1^	Ref.
AWC-K-4.2-N	0.97	0.74	3.3	−103.7	This work
AWC-Na-3-N	0.97	0.75	4.0	−90.6	This work
AWC-P-3-N	0.94	0.74	3.3	−106.7	This work
AHTC-K-4.2-N	0.95	0.74	3.6	−110.9	This work
AHTC-Na-3-N	0.95	0.78	3.9	−88.0	This work
AHTC-P-3-N	0.90	0.78	3.3	−101.8	This work
Pt/C	0.99	0.83	4.2	−86.6	This work
CNT@Co_2_-Fe_1_/FePc	0.953	0.844	3.81	-	[57]
4.8% Ce-MnO_2_/C	0.872	0.783	3.95–3.97	−90	[58]
CeGS	0.92	0.81	3.6–4	−111	[59]
NHCP-1000	0.98	0.56	~4	−72	[60]
Fe–N–C/Pd_NC_	0.97	0.87	~4	−51.1	[61]
Sr/FeNC-2	0.90	0.85	3.91	−27	[62]
Fe5-PANI/C-MCS	1.09	0.85	~4	−85.1	[63]
Co,Nb-MoS_2_/TiO_2_ HSs	0.96	0.86	3.96	−56.1	[64]
Co_3_O_4_-C_3_N_4_/rGO	0.97	0.81	3.95	−87.2	[65]
Fe–N–C 900	0.982	0.871	3.96	−71	[66]
Fe_3_C@N-CNTs/800	0.98	0.85	4	−75	[67]
Co_5_-N-C-900	0.99	0.86	4	−75	[68]
ACTP_5_@Co,N-800	1.0	0.891	3.95	−74	[69]
Co_2_P/CoP@NPC-1	0.986	0.93	3.76	−69	[70]

## Data Availability

Data are contained within the article and Supplementary Materials.

## Supplementary Materials

The following supporting information can be downloaded at: https://www.mdpi.com/article/10.3390/molecules29102238/s1, Table S1: XPS analysis of the elemental composition of investigated carbon materials: ratio of each state of C, O, N and P atoms in atomic %.
