# Supplementary material for: Thermochemical Activation of Wood with NaOH, KOH and H3PO4 for the Synthesis of Nitrogen-Doped Nanoporous Carbon for Oxygen Reduction Reaction
Source: Molecules. 2024 May 10;29(10):2238. doi: 10.3390/molecules29102238 (PMC11124516; doi:10.3390/molecules29102238)
Supplement: Supplementary file 1 [file molecules-29-02238-s001.zip › molecules-2960924-supplementary.pdf]

Table S1. XPS analysis of the elemental composition of investigated carbon materials: ratio of each state of C, O, N and P atoms in atomic %.

**Sample**	**C 1s**		**N 1s**		**O 1s**		**P 2p**	
	***E*b, eV**	**at. %**	***E*b, eV**	**at. %**	***E*b, eV**	**at. %**	***E*b, eV**	**at. %**
AHTC-K-4.2-N 82	284.3	17.98	398.5	47.11	531.1	45.61		
	284.8	36.64	399.9	24,19	532.5	34.87		
	285.3	26.77	401.1	22.26	533.6	19.53		
	286.2	15.21	402.8	6.44				
	287.3	3.40						
AWC-K-4.2-N 81	283.9	9.55	397.5	13.05	530.6	34.28		
	284.5	47.49	398.3	37.45	531.9	44.15		
	285.1	25.88	399.4	23.40	533.4	21.57		
	286.0	12.67	400.7	26.10				
	287.1	4.40						
AHTC-Na-3-N 39	284.3	17.34	398.7	48.85	530.6	14.55		
	284.7	35.84	400.4	27.08	532.2	53.00		
	285.2	28.54	401.7	16.90	533.5	25.18		
	286.1	14.75	403.6	7.17	535.4	7.26		
	287.3	3.52						
AWC-Na-3-N	282.5	11.47	398.7	46.38	529.7	34.27		
	284.7	60.98	400.2	32.44	531.5	37.81		
	285.4	14.50	401.5	21.19	533.2	27.92		
	286.2	8.30						
	287.1	4.51						
AHTC-P-3-N	284.2	6.43	396.6	4.08	528.8	21.66	131.1	39.33
	284.8	55.83	398.6	62.27	530.7	36.30	133.3	60.67
	285.7	20.09	400.2	25.53	532.5	42.04		
	286.6	12.10	401.4	8.12				
	287.7	5.54						
AWC-P-3-N 42	284.2	11.33	398.4	38.10	530.6	32.80	132.5	26.56
	284.7	48.94	399.6	35.39	532.2	49.92	133.4	47.06
	285.3	19.28	401.1	26.50	533.5	17.28	134.3	26.38
	286.3	20.45						
